# Drug development progress in duchenne muscular dystrophy

**DOI:** 10.3389/fphar.2022.950651

**Published:** 2022-07-22

**Authors:** Jiexin Deng, Junshi Zhang, Keli Shi, Zhigang Liu

**Affiliations:** ^1^ School of Nursing and Health, Henan University, Kaifeng, China; ^2^ Department of Neurology, Huaihe Hospital of Henan University, Kaifeng, China; ^3^ School of Medicine, Henan University, Kaifeng, China; ^4^ Department of Orthopedics, First Affiliated Hospital of Henan University, Kaifeng, China

**Keywords:** duchenne muscular dystrophy (DMD), drug developement, therapeutic strategies, clinical trial, research and development

## Abstract

Duchenne muscular dystrophy (DMD) is a severe, progressive, and incurable X-linked disorder caused by mutations in the dystrophin gene. Patients with DMD have an absence of functional dystrophin protein, which results in chronic damage of muscle fibers during contraction, thus leading to deterioration of muscle quality and loss of muscle mass over time. Although there is currently no cure for DMD, improvements in treatment care and management could delay disease progression and improve quality of life, thereby prolonging life expectancy for these patients. Furthermore, active research efforts are ongoing to develop therapeutic strategies that target dystrophin deficiency, such as gene replacement therapies, exon skipping, and readthrough therapy, as well as strategies that target secondary pathology of DMD, such as novel anti-inflammatory compounds, myostatin inhibitors, and cardioprotective compounds. Furthermore, longitudinal modeling approaches have been used to characterize the progression of MRI and functional endpoints for predictive purposes to inform Go/No Go decisions in drug development. This review showcases approved drugs or drug candidates along their development paths and also provides information on primary endpoints and enrollment size of Ph2/3 and Ph3 trials in the DMD space.

## 1 Introduction

Duchenne muscular dystrophy (DMD) is a severe, progressive, and incurable X-linked genetic disorder (Hoffman et al., 1987). Patients with DMD have an absence of functional dystrophin protein caused by over 7,000 mutations on the dystrophin gene, which is one of the largest human genes with 79 exons and approximately 2.4 million base pairs (Bladen et al., 2015; Carter et al., 2018). Although accounting for only 0.002% of the total muscle protein, dystrophin plays an essential role to maintain muscle functionality by linking the intracellular cytoskeleton network of muscle fiber cells to the transmemebrane components of the dystrophin-glycoprotein complex (DGC), including dystroglycan, sarcoglycans and sarcospan (Hoffman et al., 1987; Gao and McNally, 2015). Dystroglycan is composed of the α and β subunits; α-dystroglycan resides on the extracellular surface of sarcolemma, which is a specialized cell membrane surrounding striated muscle fiber cells, and is a receptor for laminin-2, linking the DGC to the extracellular matrix (ECM); whereas β-dystroglycan is a transmembrane protein tightly associated with α-dystroglycan and binds to dystrophin (Gao and McNally, 2015). The absence of dystrophin weakens the link between the cytoskeleton and sarcolemma, leading to damage of muscle fibers during contraction (Allen et al., 2016). This chronic damage results in inflammation and inhibition of muscle fiber regeneration. The subsequent replacement of muscle by fibrotic and adipose tissue eventually leads to progressive deterioration of muscle quality and loss of muscle mass.

Since dystrophin gene is on X chromosomes, mutations in the dystrophin gene lead to insufficient dystrophin production for proper muscle function in approximately 1 in 5,000 males (Mendell et al., 2012; Moat et al., 2013). If a female inherits a dystrophin mutation on one of her X chromosomes, she usually gets sufficient dystrophin from her healthy gene on the other X chromosome but will be a carrier for the disease. Clinically, symptoms of DMD initially manifest at around 2–3 years of age, followed by progressive multi-system deterioration including muscle weakness, respiratory insufficiency, musculoskeletal deformities, and cardiomyopathy (Birnkrant et al., 2018a; Birnkrant et al., 2018b; Birnkrant et al., 2018c). Wheelchair dependency usually occurs at around 12 years of age, and assisted ventilation may be needed at around 20. Cardiac issues may begin to appear as muscle weakness progresses, and symptoms may be exacerbated by lack of exercise or physical demand (Emery, 2002). Since about 40% of patients ultimately die of cardiac causes, cardiac treatment should be deployed presymptomatically for prophylaxis of cardiomyopathy (Bourke et al., 2019). The diagnosis of DMD patients may include clinical evaluations, such as patient’s detailed medical history and lab test to assess creatine kinase (CK) elevation, as well as genetic testing for dystrophin mutations (Birnkrant et al., 2018b). In cases of intronic mutatons and rearrangements, muscle biopsies will be required to test for the presence of dystrophin.

Although there is currently no cure for DMD, improvement in treatment care and management could delay disease progression and improve quality of life, thereby prolonging life expectancy for these patients. Common standard of care to address respiratory muscle weakness and decreased pulmonary function include the use of respiratory assist devices and non-invasive ventilation (NIV) (Finder et al., 2004; Lofaso et al., 2006; McKim et al., 2013). Angiotensin converting enzyme (ACE) inhibitors, angiotensin receptor blockers (ARB), and beta blockers should be used presymptomatically to prevent cardiomyopathy in DMD patients (McNally, 2007, 2017; Buddhe et al., 2018; Bourke et al., 2019). Furthermore, most patients use corticosteroids to improve muscle function and delay the loss of ambulation and onset of cardiomyopathy (Angelini et al., 1994; Biggar et al., 2004; Biggar et al., 2006; Bach et al., 2010; Schram et al., 2013; Matthews et al., 2016; McDonald et al., 2018; Zhang et al., 2021). However, corticosteroid treatment is not curative and can lead to many AEs in patients, such as weight gain, short stature, behavioral changes, osteoporosis, and bone fractures (Angelini and Peterle, 2012). Therefore, additional treatment strategies that demonstrate more effectiveness and fewer AEs than steroids are urgently needed for the treatment of DMD.

**FIGURE 1:**
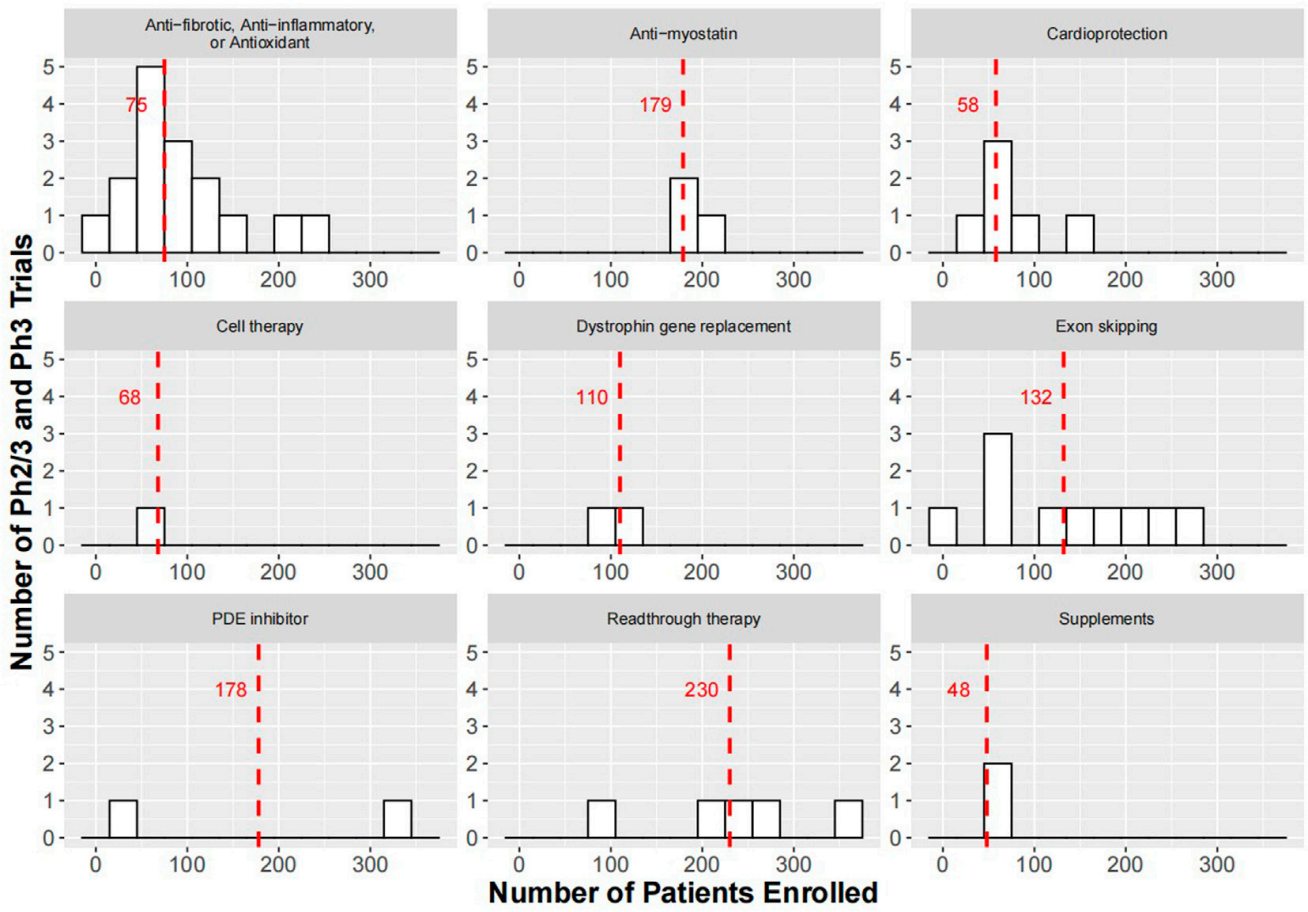
Histograms showing enrollment sizes for Ph2/3 and Ph3 trials of therapeutic strategies for the treatment of DMD. Red dashed lines indicate medium enrollment size for trials within each therapeutic strategy. For study start and anticipated study end date of the trials please refer to Supplementary Table S2. In cell therapy, there was a pivotal Ph3 study (HOPE-3, NCT05126758) with an expected completion in 2024 to evaluate the safety and efficacy of CAP-1002 in 68 DMD patients. The smaller than expected sample size may be based on the anticipated effect size and variability in the primary endpoint, change in full Performance of the Upper Limb test version 2 (PUL 2.0) at Month 12, which achieved significance in 20 participants (12 placebo and 8 treated) enrolled in the Ph2 trial (HOPE-2, NCT03406780) (Capricor, 2021).

This review discusses drugs already approved, as well as development progress made in recent years by therapeutic strategies. Information on clinical trials conducted in the DMD space are compiled in Supplementary Table S1 and discussed below to showcase approved drugs or drug candidates along their development paths. Furthermore, information on the primary endpoints and the number of patients enrolled in Ph2/3 and Ph3 trials registered on ClinicalTrials.gov are summarized in Figure 1 and Supplementary Table S2.

## 2 Gene replacement therapies

**FIGURE 2:**
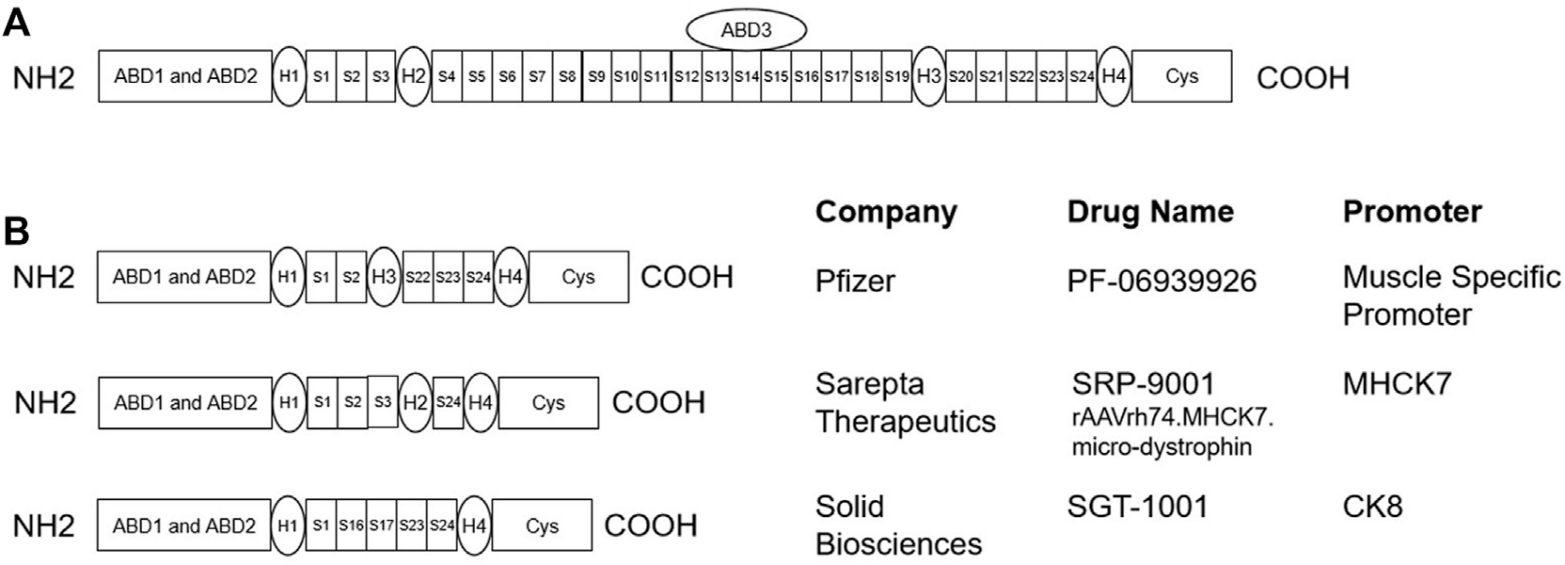
**(A)** Schematic for full length dystrophin protein (Hoffman et al., 1987): the actin-binding domains (ABD1-ABD3) bind to the intracellular cytoskeleton network of muscle cells, and the cysteine-rich (Cys) domain binds to β-dystroglycan, which is connected to the extracellular matrix protein laminin. The amino terminal and Cys domain are connected by 24 spectrin-like repeats (S1-S24) and 4 hinge domains (H1-H4). **(B)** Schematics for microdystrophin gene therapy candidates, which are truncated versions of the full length dystrophin, that are currently under development (Wang et al., 2000; Harper et al., 2002; Schneider et al., 2017).

Since DMD patients lack the dystrophin protein, the most direct treatment strategy is to replace the dystrophin gene through gene therapy. Ideally, an effective gene therapy should express dystrophin not only in the limb muscles but also in the heart and diaphragm. The recombinant adeno-associated virus (AAV) is especially suited as a vector for DMD gene therapy since most serotypes could transduce skeletal muscle with high efficiency (Duan, 2018). Since DMD gene and full-length dystrophin mRNA transcripts are exceptionally large (2.2 Mb and 14 kb, respectively), microdystrophin, which are truncated versions of dystrophin found in BMD patients, could be engineered to fit the AAV vector capacity (Figure 2). Currently, four notable gene therapy candidates are under development: PF-06939926 from Pfizer, SRP-9001 from Sarepta Therapeutics, SGT-1001 from Solid Biosciences, and GNT 0004 from Genethon. There are two Ph3 trials registered at ClinicalTrials.gov to evaluate PF-06939926 and SRP-9001. These trials have an enrollment size of 99–120, and the primary endpoints for efficacy are both change from baseline in the North Star Ambulatory Assessment (NSAA) total score at Week 52.

### 2.1 PF-06939926

PF-06939926 (fordadistrogene movaparvovec) from Pfizer delivers a microdystrophin gene under the control of a human muscle-specific promoter using AAV serotype 9 (AAV9). Currently, there is a Ph1b open-label clinical trial (NCT03362502) to investigate the safety and tolerability of PF-06939926 in DMD patients. Participants are assigned to receive either 1 × 10^14^ or 3 × 10^14^ vector genomes (vg)/kg and are followed up to 5 years after treatment (Moorehead et al., 2020). In May 2020, after dosing 3 DMD boys at the low dose (1 × 10^14^ vg/kg) and 6 at the high dose (3 × 10^14^ vg/kg), Pfizer announced that PF-06939926 had a manageable safety profile and showed preliminary effectiveness in terms of dystrophin expression and motor function (Pfizer, 2020b). In October 2020, PF-06939926 received Fast Track designation from the U.S. FDA based on data from the Ph1b study that indicated that the intravenous (IV) administration of PF-06939926 was well tolerated during the infusion period and dystrophin expression were sustained over a 12-month period (Pfizer, 2020a). In March 2021, the Ph1b study dosed 19 patients in total, and PF-06939926 continued to show efficacy and acceptable safety profile that led the team to expand the study into the non-ambulatory DMD population (Belluscio et al., 2021; Johnson, 2021). In September 2021, Pfizer reported three serious adverse events of muscle weakness, two of which involved myocarditis, in its ongoing studies, which led the company to amend the study protocol and exclude patients with specific gene mutations seen in about 15% of the DMD population (Pfizer, 2021a). In December 2021, Pfizer announced the unexpected death of a participant in the non-ambulatory arm that led FDA to place a hold on the Ph1b trial (Pfizer, 2021b). After acknowledging this, Pfizer further amended the protocol to include a 7-days hospitalization period in order to closely monitor and management patients after receiving gene therapy (Philippidis, 2022). In April 2022, FDA lifted the clinical hold on Pfizer’s investigational new drug application for the program. There is also a Ph3 trial called the CIFFREO (NCT04281485) actively enrolling 99 patients to evaluate the safety and efficacy of PF-06939926 for the treatment of DMD.

### 2.2 SRP-9001

SRP-9001 (delandistrogene moxeparvovec, rAAVrh74. MHCK7. microdystrophin) from Sarepta uses AAV serotype rh74 (AAVrh74) to deliver microdystrophin under the control of a muscle-specific promoter (MHCK7) to enhance expression in heart and skeletal muscles. According to preliminary results from the Ph1/2 study (Study 101, NCT03375164) presented at the 23rd International Congress of the World Muscle Society in October 2018, muscle biopsies from 4 boys treated with SRP-9001 (2 × 10^14^ vg/kg) showed high levels of microdystrophin protein in at least 80% of the muscle fibers after 90 days of treatment. All 4 boys showed an average raw score improvement of 6.5 points from baseline in NSAA in the first 90 days after treatment (Sarepta, 2018). The safety and tolerability data at 1 year of Study 101 was also published in JAMA Neurology (Mendell et al., 2020). In July 2020, SRP-9001 received fast track designation from the U.S. FDA (Sarepta, 2020). In May 2021, Sarepta announced that in the Ph1 ENDEAVOR study (Study 103, NCT04626674), biopsies from the first 11 boys treated with SRP-9001 at the dose of 1.33 × 10^14^ vg/kg showed acceptable safety profile with robust expression of the microdystrophin protein at Week 12: mean levels were 55.4% of normal as assessed by Western blot, and the mean percentage of dystrophin positive fibers was 70.5% as measured by immunofluorescence (Sarepta, 2021c). Topline results from Part 1 of the Ph2 trial (Study 102, NCT03769116) reported in January 2021 showed that NSAA total score in the SRP-9001 arm were statistically no better than in the placebo group (Sarepta, 2021a); however, topline results from Part 2 of the study showed that participants from the placebo crossover group score 2 points higher on the mean NSAA at Week 48 compared to the pre-specified external control cohort (Sarepta, 2022). Sarepta is also recruiting for the Ph3 EMBARK study (NCT05096221) to evaluate the safety and efficacy of SRP-9001 in 120 DMD patients.

### 2.3 SGT-1001

SGT-1001 is a gene therapy being developed by Solid Biosciences to treat DMD. Currently, there is an ongoing Ph1/2 adaptive study called IGNITE DMD (NCT03368742) to evaluate the safety and efficacy of a single IV dose of SGT-1001 in ambulatory and non-ambulatory boys with DMD. In March 2018, the study was put on clinical hold when the first patient dosed with 5 × 10^13^ vg/kg was hospitalized due to reduction in platelet and red blood cell counts and showed signs of complement activation (Solid, 2018a). The patient later recovered, and the clinical hold was subsequently removed by the FDA in June 2018 (Solid, 2018b). In February 2019, Solid Biosciences announced that 6 patients have been enrolled in the IGNITE DMD trial, 3 to the active group (5 × 10^13^ vg/kg) and 3 to the delayed treatment control group. Very low dystrophin levels (≤5%) were detected by Western blot in the 3-months biopsy samples from patients in the active group (Solid, 2019a). Based on this data, Solid is preparing to dose additional patients at higher doses. In November 2019, FDA again placed a clinical hold on the IGNITE DMD trial after the third patient in the 2 × 10^14^ vg/kg cohort experienced a serious adverse event (SAE) characterized by complement activation, thrombocytopenia, a decrease in red blood cell count, acute kidney injury, and cardio-pulmonary insufficiency (Solid, 2019b). The patient was fully recovered in December 2019, and FDA lifted the clinical hold in October 2020 after reviewing manufacturing, safety, and efficacy data (Solid, 2020). Preliminary results from the IGNITE DMD trial showed functional benefit 1.5 years post treatment in patients from the 2 × 10^14^ vg/kg cohort as assessed by NSAA, 6-Minute Walk Distance (6 MWD) and Forced Vital Capacity (FVC) (Solid, 2021). In January 2022, Solid Biosciences announced that a total of nine patients had been dosed in the IGNITE DMD trial and that dosing of additional patients was planned utilizing the updated risk mitigation strategy and second-generation manufacturing process (Solid, 2022).

### 2.4 GNT 0004

Three GNT 0004 is a gene therapy based on AAV capsid and microdystrophin under joint clinical development by Genethon and Sarepta Therapeutics. In the preclinical study, locoregional (in limb musculature) and systemic intravenous delivery of a rAAV2/8 vector expressing a canine microdystrophin (cMD1) was effective in restoring dystrophin expression and stabilizing clinical symptoms in 12 treated golden retriever muscular dystrophy (GRMD) dogs (Le Guiner et al., 2017). In April 2021, Genethon announced first patient dosed in a Ph1/2/3 gene therapy trial at I-Motion, the pediatric clinical trial platform for neuromuscular diseases at Trousseau hospital in Paris. This Ph1/2/3 trial enrolling DMD patients aged 6–10 consisted of a multicenter dose determination trial, followed by a randomized efficacy part to assess the candidate’s efficacy versus placebo. A cross-over part was also planned after 1 year following placebo treatment to allow all participant to potentially benefit from treatment. The primary efficacy endpoint was the change on the NSAA scores at 1 year. A pre-inclusion study had already started to follow the disease progression in around 100 ambulatory DMD patients aged 5-9 over a period of 3 months to 3 years. This study would help to identify DMD patients for inclusion in the Ph1/2/3 gene therapy trial.

## 3 Exon skipping

It is known that patients with BMD produce truncated versions of dystrophin and have much milder disease symptoms compared with DMD patients. Most BMD patients can live well into mid- or late adulthood, and if there are no heart or breathing problems, some patients can have a near-normal life expectancy. The exon skipping strategy to treat DMD is to use antisense oligonucleotides to bind to specific sequences in the target exon in the pre-mRNA dystrophin transcript, which leads to skipping of the exon with mutation and restores the reading frame. This will enable DMD patients to produce a partially functional BMD-like dystrophin. Newer generation of the exon skipping therapeutics involves conjugation with a peptide to increase penetration into muscle tissues for improved potency. Examples of this are SRP-5051 from Sarepta Therapeutics and PGN-EDO_51_ from PepGen, currently under clinical development. Furthermore, Audentes Therapeutis in collaboration with Nationwide Children’s Hospital in Ohio, United States is developing scAAV9. U7.ACCA, which delivers small nuclear RNA (snRNA) using AAV9 for skipping one or two copies of exon 2 in DMD patients with exon 2 duplication. Exon skipping therapies from Entrada Therapeutics, Avidity Biosciences, and Dyne Therapeutics are also under various stages of preclinical development. The drugs or candidates for exon skipping, their targeted exons in the dystrophin gene, and the percentages of DMD patients having mutations in the targeted exons are listed in Table 1. Currently, there are ten Ph2/3 or Ph3 trials registered at ClinicalTrials.gov to evaluate WVE-210201, Casimersen, Golodirsen, Vitolarsen, Eteplirsen, and Drisapersen. These trials have a medium enrollment size of 130, and the primary endpoints for efficacy are NSAA total score, Time to Stand (TTSTAND), and 6 MWD at Week 48, 96, and 104.

**TABLE 1:** Drugs or candidates for exon skipping, their targeted exons in the dystrophin gene, and the percentage of DMD patients having mutations in the targeted exons (Aartsma-Rus et al., 2009; Bladen et al., 2015; Vulin et al., 2015). The candidate drug, scAAV9.U7.ACCA, delivers small nuclear RNA (snRNA) using AAV9 for skipping one or two copies of exon 2 in DMD patients with exon 2 duplication.

Targeted exons	Candidates drugs utilizing exon skipping strategy	% Of DMD patients Having mutation
2	scAAV9.U7.ACCA	<11% (duplication)
44	BMN 044	8%
	NS-089/NCNP-02	
45	Casimersen	9%
	DS-5141b	
	BMN 045	
51	SRP-5051	13–14%
	WVE-210201	
	Drisapersen	
	Eteplirsen	
53	Viltolarsen	8%
	BMN 053	
	WVE-N531	
	Golodirsen	

### 3.1 Eteplirsen (Exondys 51^®^, AVI-4658)

In 2009, Sarepta launched a Ph1/2 clinical trial (Study 28, NCT00844597) to assess the safety and efficacy of AVI-4658 in 5–15 year-old boys with DMD who are amenable to treatment by skipping exon 51. AVI-4658 was well tolerated in all patients, and significant dose-dependent, but variable, dystrophin expression was observed (Cirak et al., 2011). In 2011, another Ph2 trial (NCT01396239) enrolled 12 boy with DMD, who received AVI-4658 at 30 and 50 mg/kg or placebo once weekly for 24 weeks, after which the placebo was switched to 30 or 50 mg/kg dosing. Results show there was a 30–50% increase in dystrophin-positive fibers in biopsies taken at 24 and 48 weeks from patients treated with eteplirsen (Mendell et al., 2013). In response to FDA’s request, the dystrophin level on biopsies taken after 180 weeks was measured by Western blot and was found just under 1% of normal, suggesting that the 30–50% positive fibers each produced only a small amount of dystrophin (Aartsma-Rus and Krieg, 2017). Sarepta then initated a Ph3 study called PROMOVI (NCT02255552) in November 2014 to provide evidence of efficacy of eteplirsen in DMD patients amenable to skipping exon 51. The study included 79 DMD patients who received eteplirsen 30 mg/kg per week for 96 weeks. In September 2016, eteplirsen received accelerated approval based on dystrophin increase in treated patients, but under accelerated approval provisions, FDA has requested further confirmation on the drug’s clinical benefit (FDA, 2016). The EMA did not approve eteplirsen after reviewing the data in 2018 (EMA, 2018). In 2021, it was reported that eteplirsen-treated patients in the PROMOVI trial showed 7-fold increase of dystrophin protein versus baseline at Week 96, with an absolute dystrophin level of only 0.63% of normal, however (McDonald et al., 2021). Comparison with historical data and previous studies indicate that treatment with eteplirsen lead to clinically meaningful improvements in lung function and 6 MWD. Currently, there is a two-part Ph3 study (MIS51ON, NCT03992430) active through 2026 to further compare the safety and efficacy of a high dose (>30 mg/kg) of eteplirsen in 154 DMD patients amenable to exon 51 skipping.

### 3.2 Golodirsen (Vyondys 53^®^, SRP-4053)

In 2015, Sarepta conducted a Ph1/2 trial (NCT0231096) to assess the safety, tolerability, efficacy, and lucocorticoids of SRP-4053 in DMD patients amenable to exon 53 skipping. The study consisted of two parts: in Part 1, 12 patients received escalating doses of SRP-4053 (4, 10, 20, and 30 mg/kg/week for at least 2 weeks) or placebo for 12 weeks; in Part 2 all patients from Part 1 plus 13 additional patients received 30 mg/kg/week for up to 168 weeks, with an additional group of untreated patients amenable to exon 53 skipping recruited as control. In 2019, it was reported that with once-weekly treatment of SRP-4053 for 48 weeks, the mean dystrophin protein level increased from 0.095 to 1.019% (Frank et al., 2020). At 3 years, patients treated with golodirsen demonstrated significant benefit in terms of 6 MWT and loss of ambulation (Servais et al., 2022). After lucocort rejecting the application over safety concerns, the FDA later reversed its decision granting golodirsen accelerated approval in 2019. The accelerated approval of golodirsen was based on the surrogate endpoint of an increase in dystrophin production in the Ph1/2 trial (NCT0231096), and further trials are required to confirm the drug’s clinical benefit (FDA, 2019). Currently, a Ph3 study (ESSENCE, NCT02500381) is actively recruiting DMD patients amenable to exon 53 skipping to evaluate the efficacy of golodirsen and casimersen. A Ph3 extension study (NCT03532542) is also active to evaluate the safety and tolerability of long-term treatment with golodirsen or casimersen.

### 3.3 Casimersen (Amondys 45^®^, SRP-4045)

A randomized placebo-controlled Ph1 study (NCT02530905) was conducted to assess the safety, tolerability, and pharmacokinetics of escalating multiple doses of SRP-4045 for 12 weeks in DMD patients with mutations amenable to exon 45 skipping. There was also an open label extension period for all participants who completed the double-blind period to receive SRP-4045 30 mg/kg once weekly for up to Week 144. The results showed that SRP-4045 was well tolerated and had little to no accumulation following dosing at 30 mg/kg (Wagner et al., 2021b). Currently, a double-blind, placebo-controlled trial (ESSENCE, NCT02500381) is ongoing to evaluate the safety and efficacy of casimersen (SRP-4045) and golodirsen (SRP-4053). Participants with mutations amenable to exon 45 or 53 skipping were randomized to receive once weekly intravenous infusions of 30 mg/kg of SRP-4045 or SRP-4053, respectively, or placebo for up to 96 weeks. Interim analysis showed significant increase from baseline in mean dystrophin levels via Western blot analysis in patients receiving casimersen for 48 weeks (1.74 vs. 0.93% of normal, *p* < 0.001) (Iannaccone et al., 2022). When compared to placebo, the mean difference in dystrophy level increase was 0.59% (*p* = 0.004). In 2021, the U.S. FDA granted accelerated approval to casimersen (Amondys 45^®^) based on interim analysis of the casimersen arm in ESSENCE trial, which demonstrated significant increase in dystrophin levels from baseline at Week 48 compared to those of the placebo arm (Sarepta, 2019). Further data is required to demonstrate clinical benefit of casimersen from the ESSENCE trial, which is anticipated to complete in April 2024.

### 3.4 Viltolarsen (Viltepso^®^, NS-065/NCNP-01)

In 2013, an exploratory Ph1 study (NCT02081625) was conducted to assess the safety, tolerability, efficacy, and pharmacokinetics in 10 Japanese DMD patients with mutations amenable to exon 53 skipping. Participants received NS-065/NCNP-01 at 1.25, 5, and 20 mg/kg once weekly for 12 weeks. Results showed that NS-065/NCNP-01 induced exon 53 skipping in a dose-dependent manner and increased dystrophin/spectrin ratio in 7 of 10 patients (Komaki et al., 2018). In a Ph2 dose-finding study (NCT02740972) conducted in 2016, 12 DMD patients on stable glucocorticoid therapy were randomized to receive 40 and 80 mg/kg NS-065/NCNP-01 weekly for 24 weeks. Additionally, 2 patients for each cohort received placebo for 4 weeks followed by treatment for 20 weeks. After 20–24 weeks of treatment, there were significant increase in dystrophin expression from baseline in both 40 (5.7 vs. 0.3%) and 80 mg/kg (5.9 vs. 0.6%) cohorts (Clemens et al., 2020). Furthermore, all participants treated with NS-065/NCNP-01 showed significant improvement in functional assessments, including time to TTSTAND, time to run/walk 10 m, and 6 MWD at Week 25 compared to matched historical control (Clemens et al., 2020). The 16 patients who participated in the Ph2 trial (NCT02740972) in U.S. and Canada were eligible to enroll in the open-label extension study (NCT03167255), where they continued to receive 40 and 80 mg/kg for an additional 192 weeks. Interim analysis at Week 109 demonstrated significant benefit in the primary endpoint, TTSTAND, over 2 years of treatment with NS-065/NCNP-01 compared to matched historical control (Clemens et al., 2022). Also, in 2016, a Ph1/2 study (Japic CTI-163291) was conducted in 16 Japanese DMD patients to evaluate the efficacy, safety, and pharmacokinetics of NS-065/NCNP-01. There was a significant increase from baseline in dystrophin expression in the 80 mg/kg cohort at Week 12 and 24 (1.18 and 5.21% vs. 0.41% of normal, respectively) (Komaki et al., 2020). In August 2020, the FDA granted accelerated approval to viltolarsen (Viltepso^®^) based on data demonstrating an increase in dystrophin production in DMD patients with mutations amenable to exon 53 skipping (FDA, 2020). Further data is required to confirm viltolarsen’s clinical benefit. Currently, a Ph3 trial called RACER53 (NCT04060199) is ongoing to evaluate the efficacy of viltolarsen, with an anticipated completion date of December 2024. The primary endpoint of RACER53 is TTSTAND at Week 48, and secondary endpoints include time to run/walk 10 m, 6 MWD, NSAA, and time to climb 4 stairs (4SC).

### 3.5 SRP-5051 (Vesleteplirsen)

In 2017, a Ph1 study (NCT03375255) was conducted to evaluate the safety, tolerability, and pharmacokinetics of SRP-5051 in DMD patients, 12 and older, with mutations amenable to exon 51 skipping. Participants received 1 of 5 single escalating doses of SRP-5051 via intravenous infusion. Plasma concentrations were measured at 0.25, 0.5, 1, 2, 4, 8, and 12 h post dose for pharmacokinetic assessment, and safety and tolerability data were collected for up to 14 weeks. In 2019, a two-part Ph2 trial (NCT04004065) was started, with Part A to evaluate the safety and tolerability in DMD patients, ages 7–21, after receiving ascending doses of SRP-5051 every 4 weeks, and Part B to further evaluate SRP-5051 doses recommended from Part A. Results at 12 weeks showed that SRP-5051 dosed monthly at 30 mg/kg increased mean dystrophin expression to 6.55%, consistently higher than the other dosing cohorts and weekly eteplirsen at 24 weeks (Sarepta, 2021b). Despite added therapeutic effectiveness, safety and tolerability concerns need to be further investigated, as there were 3 serious, treatment-emergent AEs in two patients in the 30 mg/kg cohort, including two cases of hypomagnesemia (Sarepta, 2021b; Sheikh and Yokota, 2022).

## 4 Readthrough therapy

In about 13% of patients, DMD is caused by a nonsense mutation in the dystrophin gene, causing premature halt of translation that results in production of non-functional dystrophin (Aartsma-Rus et al., 2016). The so-called readthrough therapy relies on the inhibition of translation termination by nonsense mutation, thereby restoring the production of functional dystrophin. One of the first compounds tested for this purpose both *in vitro* and *in vivo* was gentamycin (Barton-Davis et al., 1999; Howard et al., 2000; Dunant et al., 2003). However, chronic use of gentamycin is prohibited due to risks for nephrotoxicity and ototoxicity (Guan et al., 2000). Another compound under clinical development for readthrough therapy is ataluren. Currently, there are five Ph3 trials registered at ClinicalTrials.gov to evaluate the safety and efficacy of ataluren. These trials have a medium enrollment size of 219, and the primary endpoint used to assess efficacy was change from baseline in 6 MWD or slope of change in 6 MWD.

### 4.1 Ataluren (Translarna^®^)

Ataluren was assessed for clinical benefit in patients with DMD/BMD due to nonsense mutation in the dystrophin gene in a Ph2b randomized, double-blind, placebo-controlled study (NCT00592553). The primary endpoint (6 MWD) was almost met, with an observed difference of 29 m instead of the pre-specified 30 m at Week 48. However, post-hoc analysis found that in a subgroup of patients with baseline 6 MWD < 350 m, the difference between treated and control group is at least 60 m (Bushby et al., 2014). The EMA reviewed the data and concluded that the beneficial effects of ataluren were considered plausible and clinically relevant for DMD with high unmet medical need. In August 2014, a conditional approval was granted by the EMA for ambulatory patients with nonsense mutation in the dystrophin gene aged 5 years and older (Haas et al., 2015). The FDA, however, did not approve the compound. In 2017, the team reported results from a randomized, double-blind, placebo-controlled Ph3 trial (NCT01826487), which again failed to show significant change in the primary endpoint, 6 MWD at Week 48, although a significant effect of ataluren was shown in a subgroup of patients with 6 MWD between 300 and 400 m (McDonald et al., 2017). In light of this, the EMA extended the conditional approval of ataluren and requested that data from a new confirmatory trial be provided by September 2022 (EMA, 2021). After review data from the two confirmatory trials, the FDA again refused to approve the application for ataluren in October 2017. The EMA, on the other hand, adopted positive opinion to expand the indication of ataluren to include patients as young as 2 years of age in June 2018, after reviewing data from a Ph2 open-label study in DMD boys between ages 2 and 5 with a nonsense mutation in the dystrophin gene (NCT02819557) (PTC, 2018).

## 5 Cell therapy

The rationale behind cell therapy for the treatment of DMD is that precursor cells, such as stem cells, can be expanded *in vitro* and transplanted into DMD patients to repair damaged muscle. Stem cell based therapies can be based on two strategies. The first strategy is autologous stem cell transfer using cells from DMD patients that are genetically altered *in vitro* to restore dystrophin expression and are subsequently re-implanted. The second strategy is allogenic stem cell transfer using cells from an individual with functional dystrophin for transplantation into a DMD patient. In the second strategy, transplanted cells express functional dystrophin that could potentially address dystrophin deficiency in DMD patients. Allogeneic human umbilical cord mesenchymal stem cells (NCT02235844) and bone marrow-derived autologous stem cells (NCT03067831) for the treatment of DMD have been evaluated in early phases of clinical development. Other cell types such as myoblasts (early muscle cells with a single nucleus that are capable of proliferation or terminal differentiation) and cardiosphere-derived cells (CDCs), such as CAP-1002, have also been evaluated clinically. CDCs act by secreting extracellular vesicles known as exomes, which alter the expression profile of macrophages and reprogram fibroblasts, thus reversing the pro-inflammatory and -fibrotic phenotype in DMD. For sustained efficacy, treatment with CAP-1002 must be repeated every 3 months as CDCs perish over time (McDonald et al., 2022). Currently, there is one Ph3 trial registered at ClinicalTrials.gov to evaluate the safety and efficacy of CAP-1002. This trial has a planned enrollment size of 68, and the primary endpoint for efficacy assessment is mean change from baseline in full upper limb function (PUL) 2.0 at Month 12.

### 5.1 CAP-1002

A Phase 1/2 trial (HOPE-Duchenne, NCT02485938) was conducted in 2016 to evaluate the safety and tolerability of CAP-1002 in 25 male patients of at least 12 years of age with DMD-related cardiomyopathy. Intracoronary CAP-1002 was well tolerated and led to significant scar size reduction and improvement in inferior wall systolic thickening, as well as in upper limb function for up to 12 months (Taylor et al., 2019). In 2018, Capricor launched a double-blind, placebo-controlled Ph2 trial (HOPE-2, NCT03406780) to evaluate the safety and efficacy of CAP-1002. Study patients were treated with either CAP-1002 or placebo via IV delivery every 3 months. Approximately 80% of the patients were non-ambulant, and all were on stable steroid treatment. Results showed that the HOPE-2 trial met its primary endpoint of Mid-level Performance of Upper Limb (mid-PUL) v1.2, by slowing decline by 71% at month 12. Furthermore, patients treated with CAP-1002 demonstrated improvements in various measures of cardiac function and structure, suggesting clinically relevant slowing of disease progression (McDonald et al., 2022). Capricor is currently planning a pivotal Ph3 study (HOPE-3, NCT05126758) to evaluate the safety and efficacy of CAP-1002 in up to 68 DMD patients with reduced ability to walk/run.

## 6 Anti-fibrotic, anti-inflammatory, or antioxidant compounds

The loss of dystrophin leads to damage of muscle fibers during contraction and triggers multiple pathological pathways, including inflammation, fibrosis, and oxidative stress. Glucocorticoids, such as prednisone, prednisolone, and deflazacort, act as anti-inflammatory agents to attenuate the progressive muscle damage, presumably through the transrepression pathway, (i.e. inhibition of nuclear factor-κB (NF-κB) signaling) (Biggar et al., 2006; Miyatake et al., 2016; Vandewalle et al., 2018). It is to be noted that glucocorticoids likely work through other mechanisms, since compounds only targeting the inflammatory response (edasalonexent and cyclosporine) have not resulted in slower disease progression. Although glucocorticoids have been used as standard treatment for DMD, their chronic use has been associated with AEs, such as weight gain, loss of bone density and osteoporosis, stunted growth, insulin resistance, and behavioral changes, through the transactivation pathway (Schacke et al., 2002; Newton and Holden, 2007; Matthews et al., 2016). The development of novel steroids that retained the transrepression effect of glucocorticoid receptor binding without triggering tranactivation pathway associated with AEs is the therapeutic strategy for compounds such as vamorolone. Furthermore, compounds, such as pamrevlumab that block the activity of connective tissue growth factor (CTGF) or idebenone that neutralizes reactive oxidative species (ROS) in the mitochondria, could address the fibrosis and oxidative stress aspects of DMD pathology (Jaber and Polster, 2015; Richeldi et al., 2020). Currently, there are sixteen Ph2/3 and Ph3 trials registered at ClinicalTrials.gov to evaluate compounds with anti-fibrotic, anti-inflammatory, or antioxidant properties to treat of DMD. These trials have a medium enrollment size of 75. Sample sizes and the primary endpoints used in individual Ph2/3 and Ph3 trials can be found in Supplementary Table S2.

### 6.1 Vamorolone

In 2018, researchers reported on outcome of a Ph1 study (NCT02415439) to assess the safety, tolerability, and pharmacokinetics of single ascending doses (0.1–20.0 mg/kg) and multiple ascending doses (1.0–20 mg/kg/day over 14 days) of vamorolone in healthy volunteers. Vamorolone was well-tolerated at all dose levels, without any side effects of traditional glucocorticoid drugs, such as bone fragility, metabolic disturbance, and immune suppression (Hoffman et al., 2018). Vamorolone was also assessed in a 2-weeks open-label multiple ascending dose (0.25, 0.75, 2 and 6 mg/kg/day) Ph2a study (NCT02760264) in 48 steroid-naive DMD boys between ages 4 to 7. Results showed that vamorolone was well-tolerated through the highest dose of 6.0 mg/kg/day and demonstrated improved safety by biomarker assessments (Conklin et al., 2018). The patients then continued to receive the same dose for 24 weeks in an extension study (NCT02760277). The results for this study reported in 2019 showed that the 2.0 mg/kg/day group met the primary efficacy endpoint in TTSTAND over untreated natural history controls from the Cooperative International Neuromuscular Research Group (CINRG) Duchenne Natural History Study (DNHS, NCT00468832), mostly without showing evidence for AEs of glucocorticoids (Hoffman et al., 2019). Patients from the two previous Ph2 trials then continued onto a 24-months long-term extension trial (NCT03038399) at the same dose levels they received earlier. Interim results for patients receiving 2.0 or 6.0 mg/kg/day (*n* = 23) showed that vamorolone treatment was associated with improvements in run/walk 10 m velocity and climb 4 stairs velocity compared with corticosteroid-naive individuals over 18-months treatment period, with fewer AEs and no stunting of growth observed with corticosteroids (Smith et al., 2020). In April 2021, Santhera announced that the long-term extension trial (NCT03038399) had been completed and that the treatment effect was maintained over 2.5 years, equivalent to a delay of about 2 years in TTSTAND, with better safety profile over corticosteroids (Santhera, 2021a). VISION-DMD, a Ph2b trial (NCT03439670) has also been completed to evaluate the safety and efficacy of vamorolone in 121 ambulant boys with DMD. In this study, vamorolone met the primary endpoint in TTSTAND at Week 24 comparing the 6 mg/kg/day dose to placebo (Santhera, 2021c), and showed sustained efficacy across multiple endpoints over 48 weeks (Santhera, 2021b). Again, treatment was well-tolerated in 90% of subjects. Vamorolone has been granted Orphan Drug status in the US and in Europe for DMD, and has received Fast Track and Rare Pediatric Disease designations by the US FDA and Promising Innovative Medicine (PIM) status from the United Kingdom MHRA for DMD. Rolling NDA submission for vamorolone was expected to commence at the end of March 2022.

### 6.2 Idebenone

In a Ph2 double-blind, placebo-controlled trial (DELPHI, NCT00654784), positive trends of efficacy for cardiac and respiratory endpoints were demonstrated at 1 year for idebenone in 21 DMD boys between 8 and 16 years of age, with greater benefit in glucocorticoid-naive than in glucocorticoid-treated patients (Buyse et al., 2013). In a subsequent Ph3 trial (DELOS, NCT01027884) conducted in 65 DMD patients not using glucocorticoids, idebenone significantly attenuated the decline in peak expiratory flow (PEF), as well as in secondary pulmonary endpoints such as FVC and forced expiratory volume (FEV) at Week 52 (Buyse et al., 2015). In July 2016, the FDA turned down Santhera’s proposal for the approval of idebenone in DMD patients not taking concomitant lucocorticoids and requested additional data in steroid-treated patients (Santhera, 2016), and the EMA, while acknowledging the positive outcome of the Ph3 DELOS trial, invited Santhera to present additional data to further link the observed treatment effects on respiratory function outcomes to patient benefits in January 2018 (Santhera, 2018). In response to regulatory requests, a large Ph3 double-blind, placebo-controlled trial (SIDEROS, NCT02814019) was conducted to assess the efficacy of idebenone in delaying the loss of respiratory function in 255 steroid-treated DMD patients. In October 2020, however, Santhera announced the discontinuation of its Ph3 SIDEROS trial as data from an interim analysis concluded that the primary endpoint, change of FVC % predicted, is unlikely to be met. As a consequence, the Santhera will end the global development of this program to focus on progressing vamorolone for DMD (Santhera, 2020).

## 7 Myostatin inhibitors

Myostatin, also known as growth differentiation factor 8 (GDF-8), is a negative regulator of muscle fiber growth and mass by inhibiting the activation of satellite cells and the mTOR pathway, which is one of the key regulators of mass growth (Gao et al., 2013; Yoon, 2017). Myostatin acts by binding to activin type 2 receptors (ACVR2A or ACVR2B), which subsequently forms heteromeric receptor complex with type 1 receptors, activin receptor-like kinase 4 (ALK4) or ALK5 for downstream signaling (Rebbapragada et al., 2003). Drastic increase in skeletal muscle mass has been observed in mice with homozygous deletion of myostatin (McPherron et al., 1997). Similary, a hypermuscular phenotype was observed in humans, cattle, sheep, and dogs with spontaneous mutations in the myostatin gene (McPherron and Lee, 1997; Schuelke et al., 2004; Clop et al., 2006; Mosher et al., 2007). In addition, inhibition of myostatin in *mdx* mice significantly attenuated disease severity of muscular dystrophy, suggesting that myostatin may serve as a therapeutic target for the treatment of DMD (Murphy et al., 2010). Examples of anti-myostatin therapeutics that have been tested in clinical trials include the recombinant humanized monoclonal antibody (domagrozumab), the anti-myostatin adnectin (talditercept alpha), and the decoy receptor consisting of soluble form of ACVR2B fused to a human immunoglobulin G (IgG) Fc receptor (ramatercept).

### 7.1 Domagrozumab (PF-06252616)

In 2012, Pfizer conducted a Ph 1 study (NCT01616277) to investigate the safety, tolerability, pharmacokinetics and lucocorticoids of domagrozumab following IV and subcutaneous (SC) administration in healthy volunteers. The trial enrolled a total of 73 subjects to receive a range of single ascending doses between 1 and 40 mg/kg and multiple dosing of 10 mg/kg by IV administration, as well as a single 3 mg/kg dose by SC administration. Domagrozumab was well tolerated in all subjects. The most commonly reported adverse events (AEs) were headache and fatigue, respiratory tract infections, and muscle spasms (Bhattacharya et al., 2018). In 2014, a randomized, double-blind, placebo-controlled Ph2 study (NCT02310763) was initiated to evaluate domagrozumab in 121 DMD patients between 6 and 15 years of age, who were ambulatory and were taking corticosteroids for at least 6 months prior to study start. In this trial, subjects were randomly assigned to one of three groups for 2 periods of 48 weeks each. In period 1, two groups received domagrozumab, and one group received placebo. In period 2, the group that received placebo in period 1 was switched to receive domagrozumab, and the two groups that received domagrozumab in period 1 would either continue to receive domagrozumab or was switched to placebo. The study did not meet its primary efficacy endpoint of change from baseline in 4SC at Week 49, nor were there significant treatment differences in any of the secondary endpoints, except for MRI measures including thigh muscle volume (MV) and muscle volume index (MVI) (Wagner et al., 2020; 2021a). Longitudinal disease progression models were developed for the MRI endpoints and quantify their correlations with the functional endpoint 4SC. Model predictions suggest that the treatment difference in 4SC between DMD patients on TRT and PCB at the end of 96 weeks would not be clinically meaningful (<2.5 s) (Deng, 2021). In August 2018, Pfizer announced that it was terminating the Ph2 study (NCT02310763) and the open-label extension study (NCT02907619) for lack of efficacy (Pfizer, 2018).

### 7.2 Talditercept alpha (RG6206, BMS-986089, RO7239361)

In 2014, a placebo-controlled, single and multiple ascending subcutaneous dose study was conducted to evaluate the safety, tolerability, pharmacokinetics, and pharmacodynamics of BMS-986089 in healthy volunteers. There were 5 single ascending dose cohorts (*n* = 48) and 7 weekly or twice-weekly multiple ascending dose cohorts (*n* = 96). Results showed that BMS-986089 was safe and well tolerated when given as a single or multiple weekly doses of up to 180 mg and that treatment with 5 weekly doses of 45 mg or more increased thigh muscle volume and total lean body mass in healthy volunteers (Jacobsen et al., 2016). There was also a randomized open-label study conducted to compare the bioavailability of BMS-986089 after subcutaneous injection into the arm, thigh, or the stomach of healthy volunteers. In 2015, a Ph1b/2 study (THUNDERJET, NCT02515669) was conducted to evaluate the safety and tolerability of multiple ascending subcutaneous doses of BMS-986089. Patients were randomized to receive weekly injections of the drug (4–50 mg) or placebo for 24 weeks in a double-blind phase, followed by a 48-weeks open-label phase where all participants received BMS-986089. There was also an open-label extension for 228 weeks. Results showed that at 24 weeks, BMS-986089 was well tolerated with the most common AEs being mild-to-moderate injection site reactions that resolved without treatment change (Dreghici et al., 2019; Wagner et al., 2019). Treatment with BMS-986089 also resulted in dose-dependent reductions (77–97%) in free myostatin. In 2017, a Ph2/3 randomized, double-blind, placebo-controlled study (SPITFIRE, NCT03039686) was conducted to evaluate the efficacy, safety, and tolerability of BMS-986089. Participants were randomized to receive two different weekly subcutaneous doses of BMS-986089 (low or high dose) or placebo for 48 weeks. Following the 48-weeks double-blind period, all participants continued to receive either the low or high dose of BMS-986089 for 192 weeks in an open-label period. However, data from a pre-planned interim analysis of the SPITFIRE study indicated that BMS-986089 was highly unlikely to demonstrate clinical benefit in the primary endpoint of change from baseline in NSAA (Roche, 2019). Based on this Roche Genentech announced that it was discontinuing the clinical development program studying BMS-986089 in DMD.

## 8 Utrophin regulation

Utrophin is a protein that shares significant structural homology (∼80%) to dystrophin (Love et al., 1989). As a functional autosomal paralogue to dystrophin, utrophin is expressed throughout the sarcolemma in fetal muscle and is being progressively replaced by dystrophin during the development process (Clerk et al., 1993; Schofield et al., 1993). It was shown in *mdx* mouse model that dystrophin-utrophin double mutants developed more severe muscle weakness than dystrophin-only mutants and that the overexpression of utrophin appeared to prevent the development of muscular dystrophy signs, suggesting that utrophin upregulation could be used as a strategy to treat DMD (Deconinck et al., 1997; Grady et al., 1997; Tinsley et al., 1998). Ezutromid (SMT C1100) was thus identified in a high-throughput screen for small molecules that can activate utrophin transcription and was undergoing clinical development. Currently, there are no Ph2/3 or Ph3 trials registered at Clinical Trials.gov evaluating candidate drugs utilizing this strategy.

### 8.1 Ezutromid (SMT C1100)

In 2014, a Ph1b study (NCT02056808) was conducted to evaluate the safety, tolerability, and pharmacokinetics of SMT C1100 in 12 DMD patients. Patients were divided equally into three groups: Group A were given 50 mg/kg on Days 1 and 11, and 50 mg/kg bid on Days 2–10; Group B and C were given 100 mg/kg on Days 1 and 11, and 100 mg/kg bid and 100 mg/kg tid on Days 2–10, respectively. SMT C1100 was well tolerated in this study, and most patients experienced mild AEs, with no SAEs (Ricotti et al., 2016). Since previous results showed that SMT C1100 had lower exposure in DMD patients compared with healthy volunteers, which may be attributed to diet differences, another Ph1b study (NCT02383511) was conducted to evaluate the pharmacokinetics of SMT C1100 following a balanced diet including proportions of major food groups and administration of the drug with 100 ml full-fat milk. This approach improved the absorption and confirmed that, with the formulation tested (F3) and proper food instructions on balanced diet, 2500 mg of SMT C1100 BID was able to achieve concentrations adequate for modulation of utrophin expression (Muntoni et al., 2019). In 2016, a Ph2 Proof of Concept study (PhaseOut DMD, NCT02858362) was conducted to assess the activity and safety of 1,000 (F3) or 2500 mg (F6) SMT C1100 BID for at least 48 weeks in 43 DMD patients. The primary endpoints of this study were change from baseline in magnetic resonance spectroscopy (MRS) measures of leg muscles, pharmacokinetic parameters, and treatment-emergent AEs. Interim analysis at 24 weeks showed that treatment with SMT C1100 was able to reduce muscle fiber damage and decrease muscle inflammation as measured by MRS transverse relaxation time (MRS-T2) (Summit, 2018a). However, in June 2018, Summit Therapeutics announced that it was discontinuing the development of SMT C1100 since the totality of Ph2 trial data did not provide evidence that the drug had a meaningful effect on slowing DMD progression (Summit, 2018b).

## 9 Other therapeutic strategies

Therapeutic strategies for management of DMD may also include aldosterone antagonist, such as spironolactone and eplerenone. Since aldosterone acts via mineralocorticoid receptors which serves as a common pathway leading to fibrosis, it is postulated that treatment with drugs such as spironolactone and eplerenone could have anti-fibrotic effects that is most beneficial if started early in disease course (Young, 2008). Other cardioprotective drugs under investigation to improve cardiac function in DMD include beta blockers (e.g., nebivolol, metoprolol), ACE inhibitors (e.g., enalapril and lisinopril), and ARB (e.g., losartan) (Allen et al., 2013; Dittrich et al., 2019). Furthermore, phosphodiesterase 5 (PDE5) inhibitors, such as sildenafil and tadalafil, have also been tested clinically for the treatment of DMD, since it is hypothesized that these drugs may increase vascular perfusion in the skeletal muscle when motor demand increases, thereby preventing exercise-induced muscle ischemia, injury, and fatigue (Sander et al., 2000; Kobayashi et al., 2008; Kobayashi et al., 2012). The histone deacetylase (HDAC) inhibitor, givinostat, alters the epigenetic markers of histones, thereby inducing the expression of follistatin, which is associated with enhanced muscle regeneration and reduction of inflammation and fibrosis in *mdx* mice (Consalvi et al., 2013). Currently, there have been seven Ph2/3 or Ph3 trials registered at ClinicalTrials.gov to evaluate the safety and efficacy of cardioprotective drugs, one Ph3 trial to evaluate tadalafil, and two Ph2/3 or Ph3 trials to evaluate givinostat for the treatment of DMD.

### 9.1 Spironolactone and eplerenone

In a mouse model (*mdx* “het” mice) not only deficient for dystrophin but also haploinsufficient for utrophin, *utrn* ^*+/-*^, it was demonstrated that early initiation with the combination of the aldosterone receptor antagonist, spironolactone, and the ACE inhibitor, lisinopril, significantly reduced myocardial injury and improved left-ventricular (LV) circumferential strain, which is a sensitive early marker of LV systolic dysfunction (Rafael-Fortney et al., 2011). In 2015, a double-blind, randomized, noninferiority trial (NCT02354352) comparing the cardiac efficacy at month 12 of spironolactone with another aldosterone receptor antagonist, eplerenone, was initiated in 52 DMD patients on background therapy consisting of corticosteroids, ACE inhibitors or ARBs. The results showed that spironolactone added to background therapy was noninferior to eplerenone in preserving contractile function (Raman et al., 2019). A previous trial (NCT01521546) in 42 DMD patients on background therapy of ACEI or ARB has shown that after 12 months the decline in LV circumferential strain was less in those who received eplerenone than in those who received placebo (Raman et al., 2015).

### 9.2 Sildenafil and tadalafil

It has been shown in preclinical studies that treatment with sildenafil and tadalafil improved muscle blood flow and led to benefits on muscular dystrophy. For example, treatment of the *mdx* mouse model with sildenafil significantly reduced respiratory muscle weakness and fibrosis, as well as ameliorated age-related cardiac dysfunction (Adamo et al., 2010; Percival et al., 2012). Similarly, tadalafil treatment delayed the onset of cardiomyopathy in the *mdx* mouse and GRMD dog models (Hammers et al., 2016). To determine whether PDE5 inhibition can alleviate exercise-induced skeletal muscle ischemia in DMD patients, an open-label, crossover trial with tadalafil or sildenafil was initiated in 2012 to assess exercise-induced attenuation of reflex sympathetic vasoconstriction (i.e., functional sympatholysis) in 10 boys with DMD and 10 healthy-matched male controls. Results showed that sympatholysis was impaired in DMD patients despite background therapy and that treatment with tadalafil or sildenafil could alleviate this ischemia in a dose-dependent manner (Nelson et al., 2014). To further study whether sildenafil could benefit human dystrophinopathy, a double-blind, placebo-controlled Ph2 trial was conducted in 20 DMD and BMD adults with ejection fraction ≤50%. However, the study was terminated early due to a higher number of sildenafil-treated subjects worsening in cardiac function (Leung et al., 2014). Also, Eli Lilly conducted a randomized Ph3 trial to assess whether oral once-daily doses of tadalafil for 48 weeks improved ambulatory function in 331 DMD patients between 7 and 14 years who were on background therapy of glucocorticoids. However, the study did not demonstrate efficacy for tadalafil to slow the decline in 6 MWD compared with placebo through 48 weeks (Victor et al., 2017).

### 9.3 Givinostat

An open-label Ph2 study (NCT01761292) was conducted to evaluate the safety, tolerability, pharmacokinetics, effects on histology, and clinical parameters of givinostat in 20 ambulant DMD boys aged 7–10 years. Although improvements in functional tests were not observed, treatment with givinostat for at least 12 months was well tolerated and significantly increased the fraction of muscle tissue in biopsies, while reducing the amount of fibrotic tissue, tissue necrosis and fatty replacement (Bettica et al., 2016). Currently, a double-blind, placebo-controlled Ph3 study (EPIDYS, NCT02851797) is evaluating the efficacy and safety of givinostat in 179 ambulant DMD patients between 6 and 17 years of age. The primary endpoint of the trial is mean change in 4SC at Month 18, and 6MWT, time to rise from floor, NSAA, and muscle strength are being assessed as secondary endpoints. Final results of the Ph3 study are expected by the second quarter of 2022. Patients who have been previously enrolled in one of the studies evaluating givinostat can also participate in an open-label extension study (NCT03373968). Givinostat was granted the orphan drug and fast track designations by the FDA and the orphan drug designation by the EMA. In September 2020, givinostat also received the rare pediatric disease designation by the FDA.

## 10 Discussion

In recent years, active drug development progress has been made in the DMD space. These advances all contribute to better patient care in this disease, however, DMD remains an incurable disease and additional innovation is needed. Replacing dystrophin protein through gene therapy was raised as a potential treatment strategy soon after the DMD gene was identified (Dubowitz, 1989). It has been shown that in the *mdx* mouse model for DMD, at least 15–20% of the wild-type dystrophin level was effective in preventing exercise-induced muscle damage (Barton-Davis et al., 1999; van Putten et al., 2012; Godfrey et al., 2015), whereas in humans, at least 30–50% of normal was estimated to be sufficient for preventing disease progression based on dystrophin levels in asymptomatic or mildly-symptomatic Becker muscular dystrophy (BMD) patients (Neri et al., 2007; Anthony et al., 2011). However, one needs to keep in mind that, in DMD patients, the target dystrophin level from intervening later in life could be different. Several clinical trials have been conducted to evaluate the safety and efficacy of gene therapies involving AAV delivery of microdystrophin to treat DMD, including PF-06939926 from Pfizer, SRP-9001 from Sarepta, and SGT-1001 from Solid Biosciences (Figure 2). The ongoing trials demonstrate that systemic delivery of AAV to skeletal muscle is at least feasible from a manufacturing and clinical perspective. However, significant challenges remain with gene replacement strategy. For example, it is not known how much functional improvement we can expect from expressing these microdystrophin in human skeletal muscle, given that they are truncated to retain only 30–40% of the full size dystrophin (Shin et al., 2013). Although microdystrophin has been reported to improve function in the *mdx* mouse model and the GRMD dog model, it is not clear whether this efficacy can be translated into humans (Duan, 2018). Even if functional improvement is achieved to a certain degree in humans, we have to keep in mind that, since AAV is an episomal virus, the expression of microdystrophin tends to gradually decrease over time due to muscle turnover and breakdown, while development of immune response to the AAV vector prevents repeat treatment in the same patient (Vulin et al., 2012). Furthermore, concerns regarding immunogenic responses to high-dose IV administration of viral vectors remain as SAEs in IGNITE DMD (NCT03368742) from Solid Biosciences and the unexpected death of a participant in Pfizer’s Ph1b trial (NCT03362502) led FDA to place a hold on the gene therapy programs (Solid, 2018a, 2019b; Pfizer, 2021b; Mullard, 2021). Safety issues such as this highlight the need for extensive analysis of the preparations to identify and catalog potentially immunogenic vector lot components (Martinez-Navio et al., 2021). Both Solid Biosciences and Pfizer was able to continue their programs after submitting updated safety and effectiveness data, made modifications to the protocol for risk mitigation, and took steps to improve the manufacturing process (Solid, 2018b, 2020; Philippidis, 2022). Whether gene replacement strategies to treat DMD will eventually succeed and obtain regulatory approval remains to be seen.

Exon skipping utilizes antisense oligonucleotides to bind exons with mutations, thereby causing exon skipping and restoration of the reading frame. This produces a truncated dystrophin protein in DMD patients that theoretically leads to milder symptoms of muscular dystrophy similar to BMD patients. The first clinical trials employing this strategy were conducted to evaluate drisapersen and eteplirsen, which target 13–14% of DMD patients with mutations in exon 51 (Aartsma-Rus et al., 2009; Bladen et al., 2015). While drisapersen did not receive FDA approval, an accelerated approval was granted to eteplirsen (Exondys 51^®^) based on dystrophin increase in treated patients, under the condition that additional data in confirmatory trials be submitted to demonstrate clinical benefit. In this case, dystrophin quantification was used as a surrogate endpoint in DMD, and quantification methods were developed with feedback from FDA (Charleston et al., 2018). Subsequently, the FDA also gave accelerated or conditional approvals to golodirsen (Vyondys 53^®^), viltolarsen (Viltepso^®^), and casimersen (Amondys 45^®^) for ∼17% of DMD patients amenable to exon 53 and 45 skipping. Thus far, clinical trials for exon skipping approaches targeting exon 2, 44, 45, 51, and 53 have been conducted. Clinical development targeting additional exons may be unlikely due to the increasingly smaller number of patients in each sub-population. To what extent data obtained for previous antisense oligonucleotides can be applied for future exon skipping candidates is a topic that warrants further discussion with regulators (Aartsma-Rus et al., 2017).

Aside from gene replacement and exon skipping, readthrough therapy addresses the primary defect of dystrophin deficiency by reading through the nonsense mutation (stop codon), which affects ∼13% of DMD patients (Aartsma-Rus et al., 2016). The most promising drug in this regard is ataluren, which has demonstrated a bell-shaped dose-response curve with 40 mg/kg/day treated patients performing better than placebo or higher-dose group in its Ph2b study (NCT00592553) (Finkel et al., 2013; Bushby et al., 2014). Treatment effect was further demonstrated in a pre-specified subgroup of patients with a baseline 6 MWD between 300 and 400 m in a randomized, double-blind, placebo-controlled Ph3 trial (NCT01826487) (McDonald et al., 2017). Based on this and other data, ataluren has received conditional approval from the EMA but not from the FDA. Additional studies have been conducted to address some of the questions from the FDA, including one long-term study in 360 patients to confirm the benefit of ataluren on the slope of change in 6 MWD over 72 weeks (NCT03179631) and one study to assess the increase in dystrophin in muscle biopsies from treated patients at Week 40 (NCT03648827). The main disadvantage of ataluren is that this strategy only applies to DMD patients with non-sense mutation (∼13%). Also, ataluren is an expensive drug and may not be available in all European countries based on separate marketing and price negotiations.

There are ongoing research in therapeutic strategies that target the secondary pathology of DMD, rather than addressing the primary defect of dystrophin deficiency. These include compounds that reduce inflammation, fibrosis, and oxidative stress in muscle tissues of DMD patients. Glucocorticoids, such as prednisone and deflazacort, have been used to treat DMD, and their anti-inflammatory properties are thought to be associated with the inhibition of NF-κB or transrepression pathway (Biggar et al., 2006; Miyatake et al., 2016; Vandewalle et al., 2018). LucocorticoidsResearch and development for the novel dissociative steroid, vamorolone, has been discussed earlier in this review. Although vamorolone demonstrated improved safety profile over corticosteroids, weight gain has been reported as a physician-reported AE in the pharmacovigilance report in the three consecutive clinical trials of vamorolone treatment of DMD conducted by CINRG (VBP15-002 [NCT02760264]; VBP15-003 [NCT02760277]; VBP15-LTE [NCT03038399]). The incidence of weight gain in patients receiving 6.0 mg/kg/day vamorolone was 13.2%, much lower than that in patients receiving deflazacort or prednisone (Smith et al., 2020). It is to be noted that deflazacort is generally associated with less weight gain than prednisone (Bonifati et al., 2000; Angelini, 2007). Another investigative compound acting through NF-κB inhibition is edasalonexent, which is an orally-administered novel small molecule that incorporates structural elements of two known NF-κB inhibitors salicylic acid and docosahexaenoic acid (DHA, an omega-3 fatty acid) for synergistic effects (Kopp and Ghosh, 1994; Yin et al., 1998; Zwart et al., 2010; Williams-Bey et al., 2014; Vu et al., 2016). Results from a double-blind, placebo-controlled Ph3 study (PolarisDMD) in 131 DMD patients showed that at Week 52 differences between edasalonexent and placebo for NSAA total score and timed function tests were not statistically significant, although there was consistent improvement in functional declines in the edasalonexent group. Also, a pre-specified analysis demonstrated that patients younger than 6 years of age showed more robust treatment effect. Based on top-line data from PolarisDMD, Catabasis Pharmaceuticals announced their decision to stop the development of edasalonexent. Nevertheless, development to identify novel anti-inflammatory, anti-fibrotic, and anti-oxidant compounds with less side effects will no doubt continue in the future.

By inhibiting the negative regulator of skeletal muscle mass, myostatin (also known as GDF-8), myostatin inhibitors could be used to significantly increase skeletal muscle mass and attenuate disease severity in DMD. Domagrozumab is a monoclonal anti-myostatin antibody designed to neutralize myostatin and block its activity. Results from the double-blind, placebo-controlled Ph2 study (NCT02310763) failed to show statistically significant treatment effect in the primary efficacy endpoint of change from baseline in 4SC at Week 49. However, significant treatment differences were observed for MRI measures including thigh MV and MVI (Wagner et al., 2020; 2021a). As part of the primary analysis at Week 49, longitudinal disease progression models were developed for MRI endpoints (MVI and T2 relaxation time of the muscle bundle) and quantify their correlations with the functional endpoint (4SC). Model predictions suggest that the treatment difference in 4SC between DMD patients on treatment and placebo at the end of 96 weeks would not be clinically meaningful (<2.5 s) (Deng, 2021). In August 2018, Pfizer announced that it was terminating the Ph2 study (NCT02310763) and the open-label extension study (NCT02907619) for lack of efficacy (Pfizer, 2018). Longitudinal modeling approaches characterizing the progression of MRI and functional endpoints could be used for predictive purposes to inform decisions in drug development.

It is to be noted that in a preclinical discovery study in the *mdx* mouse model, vamorolone was able to reduce proinflammatory miRNAs (miR-142-5p, miR-142-3p, miR-146a, miR-301a, miR-324-3p, miR-455-5p, miR-455-3p, miR-497, miR-652) that were highly elevated in the md*x* muscle, without activating a separate group of miRNAs associated with steroid side effects. miRNA’s have been found to represent sensitive biomarkers for DMD (Fiorillo et al., 2018). Novel methodologies in longitudinal studies could enable identification of new surrogate endpoints (e.g. MRI measures, miRNA’s) in current DMD trials evaluating candidate drugs, such as vamorolone (Trifunov et al., 2020). Additionally, quick functional tests such as the GSGC assessment, which include time and grading of gait (G), climbing a set of stairs (S), rising from a chair (C), and rising from floor (Gowers’ maneuver), could help improve trial design and develop novel therapeutics to treat DMD (Angelini, 2007). The GSGC assessment is a set of simple and reproducible tests that very with age, and longitudinal analysis of GSGC score to evaluate disease progression in DMD patients could be of interests to DMD researchers, along with biomarker analysis of MRI and miRNA measurements.

Further efforts in advancing Model-Informed Drug Development (MIDD) initiatives in DMD have been carried out by non-profit partnerships such as the Duchenne Regulatory Science Consortium (D-RSC) and the Collaborative Trajectory Analysis Project (cTAP). The D-RSC is a global collaboration formed by the Critical Path Institute (C-Path) and Parent Project Muscular Dystrophy (PPMD), a nonprofit patient advocacy organization. The D-RSC database currently contains 24 integrated datasets and continues to grow (Belfiore-Oshan et al., 2022). Based on D-RSC data from 15 clinical trials, five clinical endpoints (NSAA, forced vital capacity, and the velocities of the following three timed functional tests: TTSTAND, 4SC, and 10 m walk-run time) were modeled using nonlinear mixed-effects modeling approach (Lingineni et al., 2022). The models can be used in the clinical trial simulation (CTS) platform for choosing inclusion/exclusion criteria, selecting endpoints, and optimizing other trial design considerations. Also, cTAP is a multi-stakeholder, global research coalition in DMD with a mission to understand and account for heterogeneity in longitudinal progression of DMD, as well as creating tools and insights for drug development and evaluation. Examples of work conducted by cTAP include classifying DMD patients based upon ambulatory function trajectories measured by 6 MWD, developing a prediction model for 1-year change in 6 MWD among DMD patients, and evaluating whether real-world data and natural history data could be used as external controls in DMD drug development (Goemans et al., 2016; Mercuri et al., 2016; Goemans et al., 2020).

Finally, dietary considerations to support the care of DMD patients should not be overlooked. The effects of corticosteroid treatment and decreased physical activity may increase the risk of poor mineral density in the DMD population (Davis et al., 2015). Adequate intake of food rich in calcium and vitamin D, as well as Sun exposure, may help promote bone health in boys with DMD. Weight gain is one of the common side effects of chronic steroid treatment, however many DMD patients may experience obesity even without steroid treatment (Davis et al., 2015). Reducing intake of sugar-containing beverages and high-calorie foods may decrease weight gain. It has been reported that fructose may be better utilized by dystrophic muscles (Ellis, 1980; Angelini and Tasca, 2015b). The disease progression of 14 DMD patients with high fructose diet was compared to those of 132 DMD patients with normocaloric diet (Angelini and Tasca, 2015a). Functional tests, such as Gowers maneuver, and percent MRC index of muscle strength were improved for patients on the fructose diet. Also, 18 DMD patients given branched-chain amino acids (a mixture of leucine, valine, and isoleucine, 0.4 g/kg/day) were followed for 1 year. Although these amino acids were directed to reduce the degradation of muscle protein, no significant difference was found in Gowers maneuver between groups receiving branched-chain amino acids and placebo (Angelini and Tasca, 2015b).

## 11 Conclusion

Drug development in the DMD space is advancing very rapidly. Huge progress has been made in therapeutic strategies that target the primary defect of dystrophin deficiency, such as gene replacement, exon skipping, and readthrough therapies, as well as in strategies that target the secondary pathology of DMD, such as novel dissociative steroid and myostatin inhibitors. Currently, treatment options for DMD include corticosteroids, such as Emflaza^®^ (deflazacort) and prednisone, as well as exon-skipping therapeutics approved by the FDA, such as Exondys 51^®^ (eteplirsen), Vyondys 53^®^ (golodirsen), Viltepso^®^ (viltolarsen), and Amondys 45^®^ (casimersen), and the readthrough therapy approved by the EMA, Translarna^®^ (ataluren). Despite this, DMD remains an incurable disease, and many questions and challenges remain as DMD researchers continue to innovate in this field. Novel methodologies in longitudinal modeling and simulation could enable identification of new surrogate endpoints (e.g. MRI measures), better patient selection, and improved trial design to further develop therapeutic strategies to treat DMD.
