# Supplementary material for: Drug development progress in duchenne muscular dystrophy
Source: Front Pharmacol. 2022 Jul 22;13:950651. doi: 10.3389/fphar.2022.950651 (PMC9353054; doi:10.3389/fphar.2022.950651)
Supplement: Supplementary file 1 [file DataSheet1.docx]

Supplementary Material

**Supplementary Table 1.** Trial information found on ClinicalTrials.gov with “Duchenne Muscular Dystrophy” as the search term for Condition or Disease. There were 340 trials from ClinicalTrials.gov as assessed on 3May22. Filtering out Status with “No longer available”, “Unknown status”, or “Withdrawn” leaves 299 trials. Further filtering out trials with devices, natural history trials, or trials without any therapeutic interventions leaves 163 trials. Status: 1. Completed; 2. Recruiting; 3. Active, not recruiting; 4. Enrolling by invitation; 5. Not yet recruiting; 6. Suspended; 7. Terminated.

| **Treatment Strategy** | **Phases** | **Therapeutic** | **NCT #** | **Status** | **Therapeutic** | **NCT #** | **Status** |
| --- | --- | --- | --- | --- | --- | --- | --- |
| Anti-fibrotic,  Anti-inflammatory,  or Antioxidant | 1 | TAS-205 | 02246478 | 1 | Flavocoxid | 01335295 | 1 |
|  |  | Deflazacort | 02251600 | 1 | Spironolactone \| Prednisolone | 03777319 | 3 |
|  |  | Deflazacort | 02295748 | 1 | Bocidelpar  (ASP-0367) | 04184882 | 2 |
|  |  | Metformin | 02516085 | 1 |  |  |  |
|  | 1/2 | HT-100 | 01847573 | 7 | Canakinumab | 03936894 | 2 |
|  |  | Epicatechin | 02964377 | 1 | Pentoxifylline | 00102453 | 1 |
|  |  | Edasalonexent | 02439216 | 1 | Pentoxifylline | 00243789 | 1 |
|  | 2 | CRD007 | 01540604 | 1 | Prednisone | 04322357 | 2 |
|  |  | HT-100 | 01978366 | 7 | Vamorolone \| Prednisone | 03439670 | 1 |
|  |  | HT-100 | 02525302 | 7 | Corticosteroid | 01009294 | 7 |
|  |  | Pamrevlumab | 02606136 | 3 | MNK-1411 | 03400852 | 7 |
|  |  | TAS-205 | 02752048 | 1 | Oxatomide | 00033813 | 1 |
|  |  | Prednisolone | 02167217 | 1 | Vamorolone | 03038399 | 1 |
|  |  | Vamorolone | 02760277 | 1 | Vamorolone | 02760264 | 1 |
|  |  | Vamorolone | 05185622 | 2 | Idebenone | 00758225 | 1 |
|  |  | Idebenone | 00654784 | 1 | Coenzyme Q10 | 00033189 | 1 |
|  | 2/3 | Epigallocatechin-Gallate | 01183767 | 1 | Coenzyme Q10 \| Lisinopril | 01126697 | 1 |
|  | 3 | Pamrevlumab | 04371666 | 2 | TAS-205 | 04587908 | 2 |
|  |  | Pamrevlumab | 04632940 | 2 | Prednisone | 00110669 | 1 |
|  |  | Edasalonexent | 03917719 | 7 | Prednisone \|  Deflazacort | 01603407 | 1 |
|  |  | Edasalonexent | 03703882 | 1 | Prednisone | 00004646 | 1 |
|  |  | Tamoxifen | 03354039 | 3 | Prednisone \| Coenzyme Q10 | 00308113 | 7 |
|  |  | Idebenone | 03603288 | 7 | Idebenone | 01027884 | 1 |
|  |  | Idebenone | 02814019 | 7 | Metformin \|  L-citrulline | 01995032 | 1 |
| Anti-myostatin | 1 | BLS-M22 | 03789734 | 1 |  |  |  |
|  | 1/2 | rAAV1.CMV.huFollistin344 | 02354781 | 1 | Talditercept alpha (RO7239361) | 02515669 | 7 |
|  | 2 | Ramatercept (ACE-031) | 01239758 | 7 | Domagrozumab (PF-06252616) | 02907619 | 7 |
|  |  | Ramatercept (ACE-031) | 01099761 | 7 | Domagrozumab (PF-06252616) | 02310763 | 7 |
|  | 2/3 | Talditercept alpha (RO7239361) | 03039686 | 1 |  |  |  |
| Cardioprotection  Cardioprotection | 1 | Spironolactone | 03777319 | 3 | Isosorbide Dinitrate | 01478022 | 1 |
|  |  | Rimeporide | 02710591 | 1 |  |  |  |
|  | 2 | Ifetroban | 03340675 | 2 | P-188 NF | 03558958 | 6 |
|  | 2/3 | Bisoprolol Fumarate | 03779646 | 2 | Coenzyme Q10 \| Lisinopril | 01126697 | 1 |
|  | 3 | Eplerenone \|  Spironolactone | 02354352 | 1 | Metoprolol | 05066633 | 2 |
|  |  | Nebivolol | 01648634 | 1 | Enalapril | 02432885 | 1 |
|  | NA | Eplerenone | 01521546 | 1 | Losartan \| Lisinopril | 01982695 | 1 |
| Cell Therapy | 1 | Umbilical Cord Mesenchymal Stem Cells | 02235844 | 1 | EN001 | 05338099 | 2 |
|  | 1/2 | Myoblast transplantation | 02196467 | 2 | Stem Cells | 03067831 | 2 |
|  |  | CAP-1002 | 02485938 | 1 |  |  |  |
|  | 2 | CAP-1002 | 04428476 | 4 | CAP-1002 | 03406780 | 1 |
|  | 3 | CAP-1002 | 05126758 | 5 |  |  |  |
| Dystrophin Gene Replacement | 1 | rAAV2.5-CMV-minidystrophin | 00428935 | 1 | rAAVrh74.MCK.micro-dystrophin | 02376816 | 1 |
|  |  | PF-06939926 | 03362502 | 3 | SRP-9001 | 04626674 | 4 |
|  | 1/2 | SRP-9001 | 03375164 | 3 | SGT-001 | 03368742 | 2 |
|  | 2 | SRP-9001 | 03769116 | 3 |  |  |  |
|  | 3 | PF-06939926 | 04281485 | 3 | SRP-9001 | 05096221 | 2 |
| Exon Skipping  Exon Skipping | 1 | Viltolarsen  (NS-065/  NCNP-01) | 02081625 | 1 | SRP-5051 | 03375255 | 1 |
|  |  | WVE-210201 | 03508947 | 1 | Drisapersen (PRO051) | 01128855 | 1 |
|  |  | Casimersen  (SRP-4045) | 02530905 | 1 |  |  |  |
|  | 1/2 | BMN 053 | 01957059 | 7 | Eteplirsen  (AVI-4658) | 00844597 | 1 |
|  |  | DS-5141b | 02667483 | 1 | BMN 044  (PRO044) | 01037309 | 1 |
|  |  | WVE-N531 | 04906460 | 2 | BMN 045  (PRO045) | 01826474 | 7 |
|  |  | Golodirsen  (SRP-4053) | 02310906 | 1 | scAAV9.U7.ACCA | 04240314 | 3 |
|  |  | SRP-5051 | 03675126 | 7 | NS-089/  NCNP-02 | 04129294 | 4 |
|  |  | Eteplirsen  (AVI-4658) | 00159250 | 1 |  |  |  |
|  | 2 | Viltolarsen  (NS-065/  NCNP-01) | 03167255 | 1 | Eteplirsen  (AVI-4658) | 01396239 | 1 |
|  |  | Viltolarsen  (NS-065/  NCNP-01) | 02740972 | 1 | Eteplirsen  (AVI-4658) | 02286947 | 1 |
|  |  | DS-5141b | 04433234 | 3 | BMN 044  (PRO044) | 02958202 | 7 |
|  |  | Casimersen (SRP-4045) \| Eteplirsen  (AVI-4658) \| Golodirsen  (SRP-4053) | 04179409 | 3 | BMN 044  (PRO044) | 02329769 | 7 |
|  |  | SRP-5051 | 04004065 | 2 | NS-089/  NCNP-02 | 05135663 | 3 |
|  |  | Viltolarsen  (NS-065/  NCNP-01) | 04956289 | 2 | Drisapersen (PRO051) | 01462292 | 1 |
|  |  | Eteplirsen  (AVI-4658) | 03985878 | 4 | Drisapersen (PRO051) | 01153932 | 1 |
|  |  | Eteplirsen  (AVI-4658) | 03218995 | 1 | Drisapersen | 01910649 | 7 |
|  |  | Eteplirsen  (AVI-4658) | 01540409 | 1 | Eteplirsen  (AVI-4658) | 02420379 | 1 |
|  | 2/3 | WVE-210201 | 03907072 | 7 |  |  |  |
|  | 3  3 | Casimersen  (SRP-4045) \|  Golodirsen  (SRP-4053) | 03532542 | 4 | Eteplirsen  (AVI-4658) | 03992430 | 3 |
|  |  | Casimersen  (SRP-4045) \|  Golodirsen  (SRP-4053) | 02500381 | 2 | Eteplirsen  (AVI-4658) | 02255552 | 1 |
|  |  | Viltolarsen  (NS-065/  NCNP-01) | 04768062 | 4 | Drisapersen (PRO051) | 01254019 | 1 |
|  |  | Viltolarsen  (NS-065/  NCNP-01) | 04060199 | 2 | Drisapersen (PRO051) | 01480245 | 7 |
|  |  | Drisapersen | 01803412 | 7 |  |  |  |
|  | 4 | Golodirsen  (SRP-4053) | 04708314 | 7 | Viltolarsen  (NS-065/  NCNP-01) | 04687020 | 4 |
| HADAC Inhibitor | 1/2 | Givinostat | 01761292 | 1 |  |  |  |
|  | 2/3 | Givinostat | 03373968 | 4 |  |  |  |
|  | 3 | Givinostat | 02851797 | 3 |  |  |  |
| PDE Inhibitor | 1 | Tadalafil \| Sildenafil | 01359670 | 1 | Tadalafil \| Sildenafil | 01580501 | 1 |
|  | 2 | Sildenafil | 01168908 | 7 |  |  |  |
|  | 2/3 | Tadalafil | 05195775 | 2 |  |  |  |
|  | 3 | Tadalafil | 01865084 | 7 |  |  |  |
| Readthrough Therapy | 1 | Gentamicin | 00451074 | 1 | Gentamicin | 00005574 | 1 |
|  | 2 | Ataluren (PTC124) | 03796637 | 1 | Ataluren (PTC124) | 02819557 | 1 |
|  |  | Ataluren (PTC124) | 00264888 | 1 | Ataluren (PTC124) | 01009294 | 7 |
|  |  | Ataluren (PTC124) | 00759876 | 7 | Ataluren (PTC124) | 00847379 | 7 |
|  |  | Ataluren (PTC124) | 04336826 | 2 | Ataluren (PTC124) | 00592553 | 1 |
|  |  | Ataluren (PTC124) | 03648827 | 1 |  |  |  |
|  | 3 | Ataluren (PTC124) | 01247207 | 4 | Ataluren (PTC124) | 01826487 | 1 |
|  |  | Ataluren (PTC124) | 01557400 | 1 | Ataluren (PTC124) | 02090959 | 7 |
|  |  | Ataluren (PTC124) | 03179631 | 3 |  |  |  |
|  | NA | Ataluren (PTC124) | 02369731 | 2 |  |  |  |
| Supplement | 1 | L-Arginine | 01388764 | 1 | Metformin \|  L-Arginine | 02516085 | 1 |
|  | 2 | L-Glutamine | 00296621 | 1 |  |  |  |
|  | 2/3 | Creatine Monohydrate \| Glutamine | 00016653 | 1 |  |  |  |
|  | 3 | Glutamine \| Creatine monohydrate | 00018109 | 1 | Metformin \|  L-citrulline | 01995032 | 1 |
|  | NA | EPA and DHA | 01826422 | 1 |  |  |  |
| Tissue Growth | 1 | IGF-1 | 01207908 | 1 |  |  |  |
| Utrophin Modulation | 1 | Ezutromid  (SMT C1100) | 02056808 | 1 | Ezutromid  (SMT C1100) | 02383511 | 1 |
|  | 1/2 | rAAVrh74.MCK.GALGT2 | 03333590 | 3 |  |  |  |
|  | 2 | Ezutromid  (SMT C1100) | 02858362 | 7 |  |  |  |

**Supplementary Table 2.** Ph2/3 and Ph3 trial information found on ClinicalTrials.gov. Otherwise, same search and filter conditions apply as in Supplemental Table 1. AE: Adverse Event; BOLD: Blood Oxygen Level-Dependent; CQMS: CINRG Quantitative Measurement System; FVC %p: Percent Predicted Forced Vital Capacity; LVEF%: Left Ventricular Ejection Fraction; MFM D1: Motor Function Measure Domain 1; MPI: Myocardial Performance Index; NSAA: North Star Ambulatory Assessment; PEF %p: Percent Predicted Peak Expiratory Flow; PUL: Performance of Upper Limb; QMT: Quantitative Myometry Score; SAE: Serious Adverse Event; TEAE: Treatment Emergent Adverse Event; TRF: Time to Rise from Floor; TTSTAND: Change in Time to Stand; 4SC: Time to Climb 4 Stairs; 6MWD: 6-Minute Walk Distance.

| **Treatment Strategy** | **Phases** | **Therapeutic** | **NCT #** | **Study Start** | **Study**  **End** | **Enroll #** | **Primary Endpoint** |
| --- | --- | --- | --- | --- | --- | --- | --- |
| Anti-fibrotic,  Anti-inflammatory,  or Antioxidant | 2/3 | Epigallocatechin-Gallate | 01183767 | 2010/12 | 2018/09 | 33 | Safety and Tolerability up to Month 12 |
|  |  | Coenzyme Q10 \| Lisinopril | 01126697 | 2010/02 | 2017/12 | 63 | MPI at Month 6 |
|  | 3 | Pamrevlumab | 04371666 | 2020/07 | 2022/09 | 90 | ΔPUL at Week 52 |
|  |  | Pamrevlumab | 04632940 | 2020/12 | 2023/03 | 70 | ΔNSAA at Week 52 |
|  |  | Edasalonexent | 03917719 | 2019/03 | 2020/10 | 130 | Safety and Tolerability  up to Week 104 |
|  |  | Edasalonexent | 03703882 | 2018/10 | 2020/09 | 131 | ΔNSAA at Week 52 |
|  |  | Tamoxifen | 03354039 | 2018/06 | 2024/09 | 93 | ΔMFM D1 at Week 52 |
|  |  | TAS-205 | 04587908 | 2020/11 | 2027/05 | 80 | ΔTRF at Week 52 |
|  |  | Prednisone | 00110669 | 2004/01 | 2008/02 | 64 | 1. CQMS; 2. QMT at Year 4 |
|  |  | Prednisone \| Deflazacort | 01603407 | 2013/01 | 2019/11 | 196 | 3 dimensional outcome through Month 36 |
|  |  | Prednisone | 00004646 | 1995/04 | Not listed | 20 | Not listed |
|  |  | Prednisone \| Coenzyme Q10 | 00308113 | 2007/04 | 2010/11 | 3 | 1. ΔCardiac function; 2. ΔPulmonary function at Year 1 |
|  |  | Idebenone | 03603288 | 2018/07 | 2020/11 | 161 | Safety and Tolerability |
|  |  | Idebenone | 02814019 | 2016/09 | 2020/12 | 255 | ΔFVC %p at Week 78 |
|  |  | Idebenone | 01027884 | 2009/07 | 2014/04 | 65 | ΔPEF %p at Week 52 |
|  |  | Metformin \|  L-citrulline | 01995032 | 2013/10 | 2016/03 | 47 | ΔMFM D1 subscore at Week 26 |
| Anti-myostatin | 2/3 | Talditercept alpha (RO7239361) | 03039686 | 2017/07 | 2020/04 | 166 | ΔNSAA at Week 48 |
| Cardioprotection | 2/3 | Bisoprolol Fumarate | 03779646 | 2019/01 | 2022/07 | 42 | Δleft ventricle global longitudinal strain at Month 12 |
|  |  | Coenzyme Q10 \| Lisinopril | 01126697 | 2010/02 | 2017/12 | 63 | MPI every 6 months |
|  | 3 | Eplerenone \|  Spironolactone | 02354352 | 2015/03 | 2018/05 | 52 | Left ventricular strain  at Month 12 |
|  |  | Metoprolol | 05066633 | 2021/08 | 2026/06 | 150 | ΔLVEF% at Month 24 |
|  |  | Enalapril | 02432885 | 2009/06 | 2013/06 | 76 | Myocardial fibrosis at Year 2 |
|  |  | Nebivolol | 01648634 | 2012/02 | 2021/07 | 51 | Left ventricular systolic dysfunctions at Year 5 |
| Cell Therapy | 3 | CAP-1002 | 05126758 | 2022/05 | 2025/12 | 68 | ΔPUL 2.0 at Month 12 |
| Dytrophin Gene Replacement | 3 | PF-06939926 | 04281485 | 2020/11 | 2028/09 | 99 | ΔNSAA at Week 52 |
|  |  | SRP-9001 | 05096221 | 2021/10 | 2024/11 | 120 | ΔNSAA at Week 52 |
| Exon Skipping  Exon Skipping | 2/3 | WVE-210201 | 03907072 | 2019/09 | 2020/01 | 6 | 1. ΔDystrophin level at Week 12, 22, and 46; 2. ΔNSAA at Week 48 |
|  | 3  3 | Casimersen  (SRP-4045) \|  Golodirsen  (SRP-4053) | 03532542 | 2018/08 | 2026/08 | 260 | Subjects with SAE up to Week 148 |
|  |  | Casimersen  (SRP-4045) \|  Golodirsen  (SRP-4053) | 02500381 | 2016/09 | 2024/04 | 222 | Δ6MWD at Week 96 |
|  |  | Viltolarsen  (NS-065/  NCNP-01) | 04768062 | 2021/04 | 2026/06 | 74 | Subjects with TEAE  up to Week 96 |
|  |  | Viltolarsen  (NS-065/  NCNP-01) | 04060199 | 2020/04 | 2024/12 | 74 | ΔTTSTAND at Week 48 |
|  |  | Eteplirsen  (AVI-4658) | 03992430 | 2020/07 | 2024/11 | 154 | 1. AE incidence up to Week 148; 2. ΔNSAA at Week 144 |
|  |  | Eteplirsen  (AVI-4658) | 02255552 | 2014/11 | 2019/06 | 109 | Δ6MWD at Week 96 |
|  |  | Drisapersen (PRO051) | 01254019 | 2010/12 | 2013/06 | 186 | Δ6MWD at Week 48 |
|  |  | Drisapersen (PRO051) | 01480245 | 2011/09 | 2014/03 | 233 | Δ6MWD at Week 104 |
|  |  | Drisapersen  (PRO051) | 01803412 | 2013/05 | 2016/10 | 53 | Safety and Tolerability  up to Week 48 |
| Readthrough therapy | 3 | Ataluren (PTC124) | 01247207 | 2010/11 | 2023/12 | 270 | Safety and Tolerability  up to Year 8 |
|  |  | Ataluren (PTC124) | 01557400 | 2012/05 | 2018/01 | 94 | Subjects with TEAE  up to Week 246 |
|  |  | Ataluren (PTC124) | 03179631 | 2017/07 | 2023/07 | 360 | Slope of change in 6MWD  over 72 weeks |
|  |  | Ataluren (PTC124) | 01826487 | 2013/03 | 2015/08 | 230 | Δ6MWD at Week 48 |
|  |  | Ataluren (PTC124) | 02090959 | 2014/03 | 2018/06 | 219 | Subjects with TEAE up to Week 6 |
| HDAC Inhibitor | 2/3 | Givinostat | 03373968 | 2017/10 | 2023/12 | 206 | Incidence of TEAE up to Year 1 |
|  | 3 | Givinostat | 02851797 | 2017/06 | 2022/02 | 179 | Δ4SC after 18 months |
| PDE Inhibitor | 2/3 | Tadalafil | 05195775 | 2021/12 | 2022/10 | 25 | 1. Δpost-contractile BOLD response 2. Δpost-exercise hyperemia |
|  | 3 | Tadalafil | 01865084 | 2013/09 | 2016/03 | 331 | Δ6MWD at Week 48 |
| Supplement | 2/3 | Creatine Monohydrate \| Glutamine | 00016653 | 2000/06 | 2006/12 | 48 | Muscle strength |
|  | 3 | Glutamine \| Creatine monohydrate | 00018109 | Not listed | Not listed | Not Listed | Muscle strength |
